# Author Correction: Loss of CDK4/6 activity in S/G2 phase leads to cell cycle reversal

**DOI:** 10.1038/s41586-024-07666-9

**Published:** 2024-06-10

**Authors:** James A. Cornwell, Adrijana Crncec, Marwa M. Afifi, Kristina Tang, Ruhul Amin, Steven D. Cappell

**Affiliations:** Laboratory of Cancer Biology and Genetics, Center for Cancer Research, National Cancer Institute, Bethesda, MD USA

**Keywords:** Cell-cycle exit, Single-cell imaging, Checkpoint signalling, Cellular signalling networks, Computational models

Correction to: *Nature* 10.1038/s41586-023-06274-3 Published online 5 July 2023

In the version of the article initially published, in Fig. 4b the arrow between the p107/p130 node and the E2F4/5 node was incorrectly drawn as inhibitory (⊣) when it should have been drawn as activating (→). This error has now been corrected in the HTML and PDF versions of the article.

