# Supplementary material for: Loss of CDK4/6 activity in S/G2 phase leads to cell cycle reversal
Source: Nature. 2023 Jul 5;619(7969):363–70. doi: 10.1038/s41586-023-06274-3 (PMC10338338; doi:10.1038/s41586-023-06274-3)
Supplement: Supplementary file 1 — Methods section. [file 41586_2023_6274_MOESM1_ESM.docx]

**METHODS**

**Cell culture**

Human MCF-10A (CRL-10317), MCF7 (HTB-22), U2OS (HTB-96), RPE-1 (CRL-4000), HeLa (CRM-CCl-2), and HLF (PCS-201-013) cells were obtained from ATCC. p21^-/-^ MCF-10A cells were a gift from the Meyer laboratory and originally described in^34^, RPE1 CCNA2^dd^ were a gift from the Hochegger laboratory and originally described in^28^, and U2OS CCNA2-eYFP cells and RPE-1 CCNA2-eYFP cells were a gift from the Lindqvist laboratory and originally described in^35^. MCF7 cells were authenticated by short terminal repeat (STR) analysis. No other cell line was authenticated. p21^-/-^ MCF-10A cells were used for all experiments unless otherwise stated. MCF7, U2OS, and HLFs were cultured at 37 °C in Dulbecco’s modified Eagles medium (DMEM) (Gibco, Life Technologies, Carlsbad CA, USA) containing 10% fetal bovine serum (FBS; Gibco). For mitogen removal experiments, MCF7, U2OS, and HLF cells were incubated with the above composition supplemented with 0.3% bovine serum albumin and without FBS. MCF-10A cells were cultured in phenol red-free DMEM/F12 (Invitrogen) supplemented with 5% horse serum, 20 ng/mL EGF, 10 µg/mL insulin, 500 µg/mL hydrocortisone, 100 ng/mL cholera toxin, and 1% P/S. For mitogen removal experiments, MCF-10A cells were incubated with the above composition supplemented with 0.3% bovine serum albumin and without horse serum, EGF, and insulin. RPE-1 cells were cultured in DMEM/F12 (Invitrogen) supplemented 10% FBS (RPE1 CCNA2^dd^ cells were grown in tetracycline-free FBS) and with 0.01 mg/ml hygromycin B. For mitogen removal experiments, RPE-1 cells were incubated with the above composition supplemented with 0.3% bovine serum albumin and without FBS. All tissue culture media were supplemented with 2 mM L-glutamine, 25 μg/ml streptomycin and 25 U penicillin (Gibco). Cells were cultured in a humidified atmosphere with 5% CO2 at 37 °C. Cells were routinely tested for mycoplasma.

**Constructs and stable cell lines**

CSII-pEF1a-H2B-mTurquoise, CSII-pEF1a-mCherry-Geminin(aa1-110), CSII-pEF1a-DHB-mVenus, and CSII-pEF1a-DHB-mCherry were described previously^4,15^. cDNAs for cyclin A2 fused to mCherry were cloned into the CSII-pEF lentiviral vector. MCF-10A cells were transduced with H2B-mTurqoise lentivirus for cell tracking, mCherry-Geminin lentivirus for measurement of APC/C activity, and DHB-mVenus lentivirus for measurement of CDK2 activity. U2OS cells expressing cyclin A2-eYFP from its endogenous locus were received from A. Lindqvist^35^. These U2OS cells were transduced with H2B-mTurqoise lentivirus for cell tracking and DHB-mCherry lentivirus for measurement of CDK2 activity.

**Drugs and Inhibitors**

Cells were treated with either vehicle (DMSO) or the following drugs and inhibitors at the indicated concentrations. MEK inhibitor 1 μM (Trametinib, S2673, Selleckchem, USA), CDK4/6 inhibitor 3 μM (Palbociclib, S1116 Selleckchem, USA), CDK1 inhibitor 10 μM (RO-3306, Selleckchem, USA), PLK inhibitor 1 μM (BI6727, Selleckchem, USA), Wee1 inhibitor 1 μM (MK-1775, Sigma Aldrich, USA), CDK1/2 inhibitor III 5 μM (217714, Sigma Aldrich, USA), hydroxyurea (HU) 2 mM (H8627, Sigma Aldrich, USA), thymidine 2mM (T9250, Sigma Aldrich, USA), Asunaprevir 0.3 μM (43208, Sigma Aldrich, USA), Indole-3-acetic acid 500 μM (I3750-5G-A, Sigma Aldrich, USA), doxycycline 1 μg/mL (D9891, Sigma Aldrich, USA), and neocarzinostatin (NCS) 200 ng/μL (N9162-100UG, Sigma Aldrich, USA).

**Western blotting**

Cells were harvested using trypsin, centrifuged, and washed with PBS. Cells were then lysed with Cell Lysis Buffer (CST # 9803) on ice for 30 min. Lysates were centrifuged at high speed (10,000 × g) for 30 mins and clear supernatants were transferred to new tubes. Protein concentration was measured by the BCA method (Thermo Scientific, USA, 23225). Samples were prepared in Laemmli sample buffer and either DTT or β-mercaptoethanol (M6250, Sigma Aldrich, USA). The samples were boiled at 95 °C for 5 mins and run in SDS-PAGE with Tris-Glycine (Bio-Rad, USA) running buffer. Separated proteins were transferred onto PVDF membrane with turbo-transfer buffer through the semi-dry transfer method (Bio-Rad, USA). Membranes were blocked with 2.5% milk or 3% bovine serum albumin in TBS/Tween (0.1% Tween), incubated with primary antibody overnight at 4 °C, washed with TBS/T, and were subsequently incubated with mouse or rabbit HRP conjugated secondary antibody for 1 hr at room temperature. Blots were washed with TBS/T and developed with ECL (Thermo Fisher, 34094) and imaged with a Chemi Doc (Bio-Rad). All uncropped gel source data is available in Supplementary Figure 1.

The following antibodies were used for immunoblotting in this study: Primary Antibodies include p27 (CST, #3686, 1;500), RB1 (CST, #9309, 1:500), phospho-RB (CST, #9308, 1:1000), RBL1 (CST, #89798, 1:500), phospho-RBL2 (Abcam, Ab76255, 1:1000), RBL2 (CST, #13610, 1:1000), FOXM1 (CST, #5436, 1:500), E2F4 (Thermo Scientific, MA5 11276, 1:1000), E2F5 (Invitrogen, PA5-85578, 1:500), cyclin A2 (Santa Cruz, SC-271682, 1:100), cyclin E1 (Santa Cruz, sc-247, 1:1000), CDK2 (CST, #18048,1;1000), β-Actin (Abcam, ab6276,1:10000), and vinculin (Sigma, V9131, 1:1000). Secondary Antibodies include rabbit-HRP conjugated (CST, #7074, 1:10000) and mouse-HRP conjugated (CST, #7076,1:10000).

**Immunoprecipitation**

Samples were collected using trypsin, centrifuged, and washed with PBS. Samples were then either fractionated with Activ Motif Nuclear Complex CoIP Kit (Activ Motif, 54001) or not and lysed as described previously. Protein concentrations were measured by BCA assay. 300-500 μg of protein was incubated with of the manufacturer’s recommended amount of primary antibody overnight at 4 °C in 200 μl of Activ Motif Nuclear Complex CoIP Kit’s Low IP Buffer, or in cell lysis buffer. The following day, this antibody and cell lysate mixture was then incubated with 20μl of Protein A or G Magnetic DynaBeads for 4 hrs at 4 °C. The beads were washed three times with washing buffer (10 mM Tris/Cl pH 7.5; 150 mM NaCl; 0.25% NP-40; 0.5mM EDTA) using a magnetic rack and were eluted from the beads using Laemmli buffer and DTT or β-mercaptoethanol and then boiled. The sample was then processed as described above through western blotting. The following antibodies were used for immunoprecipitation in this study: CDK2 (CST, 18048, 1:100), E2F4 (CST, 40291,1;1000), RBL1 (CST, 89798, 1:1000), and normal rabbit IGG (CST, 2729, 1:2000).

#### **Senescence-associated β-galactosidase assay**

The cells were treated with the indicated drug conditions for 7 days then fixed in 4% paraformaldehyde for 10 mins at room temperature, washed three times in PBS, and then stained for fluorescent β-galactosidase using the SPiDER-βgal kit (Dojindo, #SG04-01) following manufacturers protocol in 24-well dishes. Fluorescent β-galactosidase activity was imaged in the GFP channel.

**Immunofluorescence**

Cells were fixed in 4% paraformaldehyde for 10 mins at room temperature, washed three times in PBS, and then blocked and permeabilised for 1 hour at room temperature with PBS containing 10% FBS, 1% bovine serum albumin, 0.1% triton, and 0.01% sodium azide. Cells were then incubated with anti-phospho-Rb (807/811) (Alexa Fluor 647 conjugate) (Cell Signalling Technology, #8974, 1:2000) for 1 hr at room temperature, or overnight at 4 °C with anti-cyclin A2 (Santa Cruz Biotechnology, sc-271682, 1:500), anti-cyclin E1 (Santa Crux, sc-247, 1:500) or anti-phospho-Histone H2A.X (γH2AX, CST, #9718, 1:1000). Non-conjugated primary antibodies were visualised using goat anti-rabbit secondary antibody, Alexa Fluor 647 or goat anti-mouse secondary antibody, Alexa Fluor 647 (Invitrogen, A-212441 or A-21245, 1:1000).

**mRNA FISH**

Cells were fixed in 4% paraformaldehyde for 10 mins at room temperature and then washed three times with PBS. RNA FISH was then carried out using the ViewRNA ISH Cell Assay kit (Thermo Fisher, QVC0001) following the manufacturer’s instructions using the following probes: CCNA2 (Thermo Fisher, VA6-15304-VC) and E2F1 (Thermo Fisher, VA6-3168356-VC).

**siRNA transfection**

Cells were transfected with siRNAs using Dharmafect 1 (Horizon Discovery, Ltd, T-2001-03) according to the manufacturer’s instructions. The following On-Target plus pooled sets of four siRNAs were used: p27 (L-003472-00-0005), FOXM1 (L-009762-00-0005), FOXO3A (L-003007-00-0005), RB1 (L-003296-02-0005), RBL1 (L-003298-00-0005), RBL2 (L-003299-00-0005), E2F4 (L-003262-00-0005), E2F5 (L-003773-00-0005). On-Target plus control siRNA (nontargeting, Dharmacon) were used as a control. The following siGENOME pooled sets of four siRNAs were used: CCNA2 (M-003205-02-0005). All siRNAs were used at a concentration of 20 nM unless noted. Cells were incubated with siRNAs for 24 hrs before being treated, unless otherwise indicated.

**qRT-PCR**

To perform gene expression analysis MCF-10A cells were seeded in 24-well plates and allowed to attach overnight. Cells were then transfected and incubated with siRNA for 24 hrs before being treated with a CDK1i overnight to enrich for post-R cells. The following day cells were treated with a CDK4/6i for 2 hrs before being lysed with RLT lysis buffer containing 1% beta-mercaptoethanol. RNA was isolated from cultured cells using RLT RNeasy 96 Qiacube HT Kit (Qiagen, 74171) and on-column DNA digest (Qiagen, 79254). The concentration of RNA was determined using a NanoDrop. Complementary DNA synthesis was done using iScript cDNA synthesis kit (Biorad) according to manufacturer’s protocol. Real-time PCR analysis was conducted using iQ SYBR Green supermix (Biorad). GAPDH was used as internal controls.  Relative expression was calculated based on the threshold cycle with the different efficiency of each primer by the 2^−ΔΔCT^ (“delta-delta CT”) method. The following primer sequences were used: CCNA2 (forward: AGTGATGTTGGGCAACTCTGC, and reverse: AGGGGTGCAACCCGTCT, purchased from Integrated DNA Technologies), CCNE1 (forward: CCCATCATGCCGAGGGAG, and reverse: TATTGTCCCAAGGCTGGCTC, purchased from Integrated DNA Technologies), E2F1 (forward: GAGGAGACCGTAGGTGGGAT, and reverse: ACAACAGCGGTTCTTGCTCC, purchased from Integrated DNA Technologies), and GAPDH (forward: GTGAAGGTCGGAGTCAACG and the reverse: TGAGGTCAATGAAGGGGTC purchased from Qiagen).

**Measurement of cyclin A2 half-life**

Cells were seeded on a 96-well plate at the density of 50,000 cells per well. Cells were synchronised with 10 μM RO3306 (CDK1i) treatment for 24 hrs and then treated with 100 μg/ml cycloheximide for 2, 4, 6, 8, 10, and 12 hrs. The cells were then fixed with 4% paraformaldehyde. The sample was then processed as described above through immunofluorescence.

**Time lapse microscopy**

Indicated cells were seeded in a 96-well plate (Ibidi, #89626­) 18 hrs prior to imaging in full growth media. Cells were seeded such that cell density remained sub-confluent until the end of the imaging period. Time-lapse imaging was performed in 300 μL full growth media. Images were taken in CFP, YFP, and RFP channels every 12 min on a Nikon Ti2-E inverted microscope (Nikon) with a 20X 0.45NA objective. We used NIS Elements (Nikon, v5.11.00) software for image acquisition. Total light exposure time was kept under 600 msec for each time point. An environmental chamber surrounding the microscope maintained cells at 37 °C with 5% humidified CO2.

**Image analysis**

All image analyses were performed with custom MATLAB scripts as previously described^15^. Cells were segmented for their nuclei based on either Hoechst staining (fixed-cell imaging) or H2B-mTurquoise (live-cell imaging). To measure CDK2 activity the cytoplasm was sampled by expanding a ring outside the nucleus (with inner radius of 0.65 μm and outer radius of 3.25 μm) without overlapping with cytoplasm from a neighboring cell. Nuclear immunofluorescence, nuclear DHB-mVenus, and nuclear mCherry-Geminin signals were calculated as median nuclear intensity, as these signals were often excluded from the nucleoli. Cytoplasmic DHB-mVenus signal were calculated as the median intensity within the cytoplasmic ring, excluding pixel intensities indistinguishable from background. Whole-cell mRNA FISH puncta and fluorescent SA β-gal measurements were made by generating a whole-cell mask by expanding the nucleus mask up to 3x the nucleus radius using the MATLAB function *bwmorph* and quantifying the total number of pixels above background within this mask.

**Single cell tracking**

Segmentation and tracking of cells from time-lapse microscopy films was performed using a previously published MATLAB pipeline^15^; code available at https://github.com/scappell/Cell_tracking. Briefly, cell tracks were linked by screening the nearest future neighbor using segmented cell nuclei in adjacent frames. When the imaging plate was removed from and put back on the microscope stage (for drug addition or drug wash off), the plate jitter was calculated by registering images of the nucleus-stained channel and corrected prior to tracking.

**Monte Carlo Simulation**

Pre-competition time-series distributions for the mitosis clock of untreated cells (measured as the time elapsed between APC/C inactivation and anaphase) and for the cell cycle exit clock (measured as the time elapsed between drug treatment and CDK2 activity falling below a threshold of 0.6) for cells treated with a CDK1i+CDK4/6i were measured by live-cell imaging and single cell tracking. Normal and lognormal distributions were fitted to the measured mitosis and cell cycle exit clock times, respectively, using the MATLAB function *fitdist*. Using these fitted parameters 10,000 data points were generated for the mitosis and cell cycle exit distribution using the *normrnd* and *lognrnd* functions, respectively, and the *ecdf* function was used to normalise the data between 0 and 1. To simulate a competition between independent competing processes 10,000 pseudorandom values were drawn from the standard uniform distribution using the *rand* function which were used to randomly sample (with replacement) from the normalised mitosis and cell cycle clock distribution. For a given simulated cell, whether mitosis or cell cycle exit won the competition was determined by calculating the difference between each pair of randomly selected mitosis and cell cycle exit clock times. This enabled for the relative frequency of cell cycle exit to be calculated as well as the distribution of cell cycle exit times.

**ODE model of the Restriction Point**

We used a previously described mathematical model of the Restriction Point pathway published by Yao et al.^8^. We modified the original model to represent the signalling architectures for the feedback loop shown in Fig. 4a and the feedforward pathway shown in Fig. 4b. The sets of differential equations for each signalling architecture are shown below:

*Feedback Loop Model:*

1. $\frac{dMEK}{dt}=\frac{1}{\tau_{MEK}}(\frac{s^{n_{1}}}{k_{1}^{n_{1}}+s^{n_{1}}}-MEK)$
2. $\frac{dCDK4}{dt}=\frac{1}{\tau_{CDK4}}(\frac{{MEK}^{n_{2}}}{k_{2}^{n_{2}}+{MEK}^{n_{2}}}-CDK4)$
3. $\frac{dRb}{dt}=\frac{1}{\tau_{Rb}}((\frac{k_{3}^{n_{3}}}{k_{3}^{n_{3}}+{CDK4}^{n_{3}}})(\frac{k_{8}^{n_{8}}}{k_{8}^{n_{8}}+{CDK2}^{n_{8}}})-Rb)$
4. $\frac{dE2F}{dt}=\frac{1}{\tau_{E2F}}(\frac{k_{4}^{n_{4}}}{k_{4}^{n_{4}}+{Rb}^{n_{4}}}-E2F)$
5. $\frac{dCCNA}{dt}=\frac{1}{\tau_{CCNA}}(\frac{{E2F}^{n_{5}}}{k_{5}^{n_{5}}+{E2F}^{n_{5}}}-CCNA)$
6. $\frac{dCycA}{dt}=\frac{1}{\tau_{CycA}}(\frac{{CCNA}^{n_{6}}}{k_{6}^{n_{6}}+{CCNA}^{n_{6}}}-CycA)$
7. $\frac{dCDK2}{dt}=\frac{1}{\tau_{CDK2}}(\frac{{CycA}^{n_{7}}}{k_{7}^{n_{7}}+{CycA}^{n_{7}}}-CDK2)$

*Parameters for Feedback Loop Model:*

| k_1_=1.4 | n_1_=3 | τ_MEK_=0.5 |
| --- | --- | --- |
| k_2_=0.3 | n_2_=2 | τ_CDK4_=0.5 |
| k_3_=0.3 | n_3_=3 | τ_Rb_=0.5 |
| k_4_=0.1 | n_4_=3 | τ_E2F_=0.5 |
| k_5_=0.1 | n_5_=2 | τ_CCNA_=0.5 |
| k_6_=0.3 | n_6_=2 | τ_CycA_=0.5 |
| k_7_=0.3 | n_7_=2 | τ_CDK2_=0.5 |
| k_8_=0.1 | n_8_=3 |  |

*Initial conditions for feedback loop model:*

pre-R state: MEK=0, CDK4=0, pPP=1, Rb=1, E2F=0, CCNA=0, CycA=0, CDK2=0

post-R state: MEK=1, CDK4=1, pPP=0, Rb=0, E2F=1, CCNA=1, CycA=1, CDK2=1

*Feedforward Model:*

1. $\frac{dMEK}{dt}=\frac{1}{\tau_{MEK}}(\frac{s^{n_{1}}}{k_{1}^{n_{1}}+s^{n_{1}}}-MEK)$
2. $\frac{dCDK4}{dt}=\frac{1}{\tau_{CDK4}}(\frac{{MEK}^{n_{2}}}{k_{2}^{n_{2}}+{MEK}^{n_{2}}}-CDK4)$
3. $\frac{dpPP}{dt}=\frac{1}{\tau_{pPP}}(\frac{{CDK4}^{n_{3}}}{k_{3}^{n_{3}}+{CDK4}^{n_{3}}}-PP)$
4. $\frac{dCCNA}{dt}=\frac{1}{\tau_{CCNA}}(\frac{{pPP}^{n_{4}}}{k_{4}^{n_{4}}+p{PP}^{n_{4}}})(\frac{{E2F}^{n_{9}}}{k_{9}^{n_{9}}+{E2F}^{n_{9}}})-CCNA)$
5. $\frac{dCycA}{dt}=\frac{1}{\tau_{CycA}}(\frac{{CCNA}_{5}^{n_{5}}}{k_{5}^{n_{5}}+{CCNA}^{n_{5}}}-CycA)$
6. $\frac{dCDK2}{dt}=\frac{1}{\tau_{CDK2}}(\frac{{CycA}^{n_{6}}}{k_{6}^{n_{6}}+{CycA}^{n_{6}}}-CDK2)$
7. $\frac{dpRb}{dt}=\frac{1}{\tau_{pRb}}(\frac{{CDK2}^{n_{7}}}{k_{7}^{n_{7}}+{CDK2}^{n_{7}}})-pRb)$
8. $\frac{dE2F}{dt}=\frac{1}{\tau_{E2F}}(\frac{{pRb}^{n_{8}}}{k_{8}^{n_{8}}+{pRb}^{n_{8}}}-E2F)$

*Parameters for feedforward model:*

| k_1_=1.4 | n_1_=3 | τ_MEK_=0.5 |
| --- | --- | --- |
| k_2_=0.3 | n_2_=2 | τ_CDK4_=0.1 |
| k_3_=0.1 | n_3_=5 | τ_pPP_=0.1 |
| k_4_=0.1 | n_4_=2 | τ_CCNA_=2 |
| k_5_=0.06 | n_5_=2 | τ_CycA_=2.5 |
| k_6_=0.1 | n_6_=1 | τ_CDK2_=0.2 |
| k_7_=0.4 | n_7_=3 | τ_pRb_=0.1 |
| k_8_=0.4 | n_8_=2 | τ_E2F_=0.1 |
| K_9_=0.1 | N_9_=2 |  |

*Initial conditions for feedforward model:*

*pre-R state:* MEK=0, CDK4=0, Rb=1, E2F=1, CCNA=0, CycA=0, CDK2=0

*post-R state:* MEK=1, CDK4=1, Rb=0, E2F=1, CCNA=1, CycA=1, CDK2=1

Using similar notation to Yao et al^8^: S = mitogens (e.g. serum), MEK = MEK activity, CDK4 = cyclin D/CDK4/6 activity, Rb = hypo-phosphorylated Rb (active Rb), E2F = E2F1-3 transcription factors, CDK2 = CDK2 activity, pPP = hyper-phosphorylated p107 and p130 (inactive forms), CCNA = cyclin A2 mRNA, CycA = cyclin A2 protein, pRb = hyper-phosphorylated Rb (inactive form). Parameters for each model were selected from the same range of parameters originally published by Yao et al.^8^.

Each system of ODEs was solved in R using the *ode* function (from the *deSolve* R package) using the LSODA algorithm. To model the effect of mitogen removal on post-R cells for the feedback loop the initial conditions of the model were set for pre-R cells, as listed above, and the model was solved for S = 0. The result is shown in Fig. 4c. To model the effect of mitogen removal on post-R cells for the feedforward pathway the initial conditions of the model were set for post-R cells, as listed above, and the model was solved for S = 0. The result is shown in Fig. 4d.

To model how varying mitogen concentration affected the steady state CDK2 activity of pre-R cells given the feedback loop vs feedforward model the initial conditions were set for pre-R cells for each model, as listed above, and the model was solved for different concentrations of mitogens (S varying from 0.01-10) over a time period of 4 hrs (Fig. 4e). To model how varying mitogen concentration affected the steady state CDK2 activity of post-R cells given the feedback loop vs feedforward model the initial conditions were set for post-R cells for each model, as listed above, and the model was solved for different concentrations of mitogens (S varying from 0.01-10) over time periods ranging from 4-24 hrs (Fig. 4e).

**Hysteresis analyses**

Our ODE model results and experimental data suggest a lack of hysteresis in post-R cells with respect to mitogen signalling. To test for hysteresis in pre- and post-R cells we treated them with different doses of a MEK inhibitor and measured the fraction of cells that exited the cell cycle (loss of CDK2 activity) as measured at different times after treatment (4, 10, 15, and 24 hrs) by live-cell imaging. All cells were pre-imaged to establish the history of each cell, and then cells were treated with a MEK inhibitor (Trametinib, S2673, Selleckchem, USA) at doses ranging from 0.01 nM to 100 nM. After treatment, cells were continuously imaged for more than 24 hours. To determine cell cycle status, CDK2 activity was measured using the CDK2 reporter as described above.

To identify the effect of MEK inhibition on pre-R cells we treated pre-R cells with various concentrations of a MEK inhibitor. In MCF-10A cells, MEK inhibition does not lead to cell cycle arrest when added in G1 phase^9^, indicating these cells are born post-R. Therefore, in our analysis we selected daughter cells whose mother had received the MEK inhibitor between 0 and 6 hours before mitosis. These daughter cells were considered as pre-R cells and the fraction of cells at each dose of MEK inhibitor that had exited the cell cycle (low CDK2 activity) 4 hours after mitosis was plotted. Additional time points after MEK inhibitor treatment were not plotted for pre-R cells since 4hrs after mitosis was sufficient for nearly all cells to exit the cell cycle and generate a clear dose-response curve.

To identify the effect of MEK inhibition on post-R cells we treated post-R cells with a combination of MEK inhibitor and a CDK1 inhibitor (to prevent the cells entering mitosis). We then measured the fraction of these cells that had exited the cell cycle (low CDK2 activity) at 4, 10, 15, and 24 hours after treatment. The same proportion of post-R cells will exit the cell cycle at the same dose of MEK inhibitor as pre-R cells, if given sufficient time (comparing blue line vs grey line). This analysis provides evidence for a lack of hysteresis in CDK2 activity with respect to mitogen signalling since both pre-R and post-R cells will lose CDK2 activity at the same dose of MEK inhibitor if given enough time to reach steady state.

**Cyclin A2 destabilization**

RPE-1 CCNA2^dd^ cells were a gift from Helfrid Hochegger (University of Sussex) and their construction was described in detail previously^27^. Briefly, the DNA encoding an auxin-inducible degron (AID) tag and the small molecule-assisted shutoff (SMASh) tag were added in tandem to the C-terminus of the CCNA2 locus in hTERT immortalized RPE1 cells (ATCC, CRL-4000) using CRISPR/Cas9. The AID system is derived from plants and is ubiquitinated by the plant F-box protein TIR1. Therefore expression of OsTIR1 (derived from *Oryza sativa*) is required for AID degradation. A TET-inducible OsTIR1 was integrated into the ROSA26 locus using CRISPR-mediated gene targeting. The resulting cell line is called RPE-1 CCNA2^dd^.

In the absence of any drugs the cyclin A2-dd protein is controlled as normal throughout the cell cycle. However, when a drug cocktail of doxycycline, indole-3-acetic acid (plant hormone in the auxin class), and asunaprevir (DIA) is added, cyclin A2-dd is destabilized and rapidly degraded. Specifically, doxycycline induces the expression of OsTIR1, indole-3-acetic acid recruits cyclin A2-dd to OsTIR1 for ubiquitination, and asunaprevir (ASV), a viral protease inhibitor, prevents SMASh tag self-cleavage, destabilizing cyclin A2-dd. The combined effect of the AID and SMASh tags ensures rapid and complete degradation of cyclin A2-dd upon addition of DIA.

**Statistical analyses**

MATLAB (Mathworks) and Prism (GraphPad Software) were used for all statistical analyses. Details of the statistical tests used are included in the figure legends.

**REFERENCES**

34 Bachman, K. E. *et al.* p21(WAF1/CIP1) mediates the growth response to TGF-beta in human epithelial cells. *Cancer Biol Ther* **3**, 221-225, doi:10.4161/cbt.3.2.666 (2004).

35 Akopyan, K. *et al.* Assessing kinetics from fixed cells reveals activation of the mitotic entry network at the S/G2 transition. *Mol Cell* **53**, 843-853, doi:10.1016/j.molcel.2014.01.031 (2014).
