# Supplementary material for: Loss of CDK4/6 activity in S/G2 phase leads to cell cycle reversal
Source: Nature. 2023 Jul 5;619(7969):363–70. doi: 10.1038/s41586-023-06274-3 (PMC10338338; doi:10.1038/s41586-023-06274-3)
Supplement: Supplementary file 2 — Reporting Summary [file 41586_2023_6274_MOESM2_ESM.pdf]

nature portfolio

Corresponding author(s): Steven D Cappell

Last updated by author(s): May 19, 2023

Reporting Summary

Nature Portfolio wishes to improve the reproducibility of the work that we publish. This form provides structure for consistency and transparency in reporting. For further information on Nature Portfolio policies, see our Editorial Policies and the Editorial Policy Checklist.

Statistics

For all statistical analyses, confirm that the following items are present in the figure legend, table legend, main text, or Methods section.

n/a	Confirmed	
☐	☒	The exact sample size (*n*) for each experimental group/condition, given as a discrete number and unit of measurement
☐	☒	A statement on whether measurements were taken from distinct samples or whether the same sample was measured repeatedly
☐	☒	The statistical test(s) used AND whether they are one- or two-sided<br>*Only common tests should be described solely by name; describe more complex techniques in the Methods section.*
☒	☐	A description of all covariates tested
☒	☐	A description of any assumptions or corrections, such as tests of normality and adjustment for multiple comparisons
☐	☒	A full description of the statistical parameters including central tendency (e.g. means) or other basic estimates (e.g. regression coefficient) AND variation (e.g. standard deviation) or associated estimates of uncertainty (e.g. confidence intervals)
☐	☒	For null hypothesis testing, the test statistic (e.g. *F*, *t*, *r*) with confidence intervals, effect sizes, degrees of freedom and *P* value noted<br>*Give P values as exact values whenever suitable.*
☒	☐	For Bayesian analysis, information on the choice of priors and Markov chain Monte Carlo settings
☒	☐	For hierarchical and complex designs, identification of the appropriate level for tests and full reporting of outcomes
☒	☐	Estimates of effect sizes (e.g. Cohen's *d*, Pearson's *r*), indicating how they were calculated

Our web collection on statistics for biologists contains articles on many of the points above.

Software and code

Policy information about availability of computer code

Data collection	NIS Elements (v5.11.00)
Data analysis	MATLAB (vR2020b). Automated image analysis was performed using custom MATLAB scripts as described in Cappell, S.D. et al Cell 166, 167-180 (2016) (https://github.com/scappell/Cell_tracking). Graphpad Prism 9 (v9.2.0) was used for statistical analysis. RStudio (v1.3.1093) was used for mathematical modeling along with the deSolve package.

For manuscripts utilizing custom algorithms or software that are central to the research but not yet described in published literature, software must be made available to editors and reviewers. We strongly encourage code deposition in a community repository (e.g. GitHub). See the Nature Portfolio guidelines for submitting code & software for further information.

Data

Policy information about availability of data

All manuscripts must include a data availability statement. This statement should provide the following information, where applicable:

- Accession codes, unique identifiers, or web links for publicly available datasets
- A description of any restrictions on data availability
- For clinical datasets or third party data, please ensure that the statement adheres to our policy

All data is available in the Source Data file. The datasets generated during and/or analyzed during the current study are also available from the corresponding author on reasonable request. All data supporting the findings of this study are available from the corresponding author on reasonable request.

Human research participants

Policy information about studies involving human research participants and Sex and Gender in Research.

Reporting on sex and gender	N/A
Population characteristics	N/A
Recruitment	N/A
Ethics oversight	N/A

Note that full information on the approval of the study protocol must also be provided in the manuscript.

Field-specific reporting

Please select the one below that is the best fit for your research. If you are not sure, read the appropriate sections before making your selection.

☒ Life sciences ☐ Behavioural & social sciences ☐ Ecological, evolutionary & environmental sciences

For a reference copy of the document with all sections, see nature.com/documents/nr-reporting-summary-flat.pdf

Life sciences study design

All studies must disclose on these points even when the disclosure is negative.

Sample size	Sample size was determined based on general standards for biological studies and requirements for statistical analysis, attempting to have a minimum of n=3 biological replicates with sufficient reproducibility.
Data exclusions	No data was excluded form the experiments.
Replication	All experiments in which p-values are present have been carried out with at least 3 replicates. All experiments were independently reproduced at least twice.
Randomization	Samples were allocated randomly for imaging and analysis. Representative single-cell traces where chosen at random from the population for visualization.
Blinding	Blinding was not relevant to this study. Image acquisition and analysis was conducted using automated scripts which are not subject to experimental bias. For western blots, blinding is not possible because samples need to be loaded in a particular order.

Reporting for specific materials, systems and methods

We require information from authors about some types of materials, experimental systems and methods used in many studies. Here, indicate whether each material, system or method listed is relevant to your study. If you are not sure if a list item applies to your research, read the appropriate section before selecting a response.

Materials & experimental systems

n/a	Involved in the study	
☐	☒	Antibodies
☐	☒	Eukaryotic cell lines
☒	☐	Palaeontology and archaeology
☒	☐	Animals and other organisms
☒	☐	Clinical data
☒	☐	Dual use research of concern

Methods

n/a	Involved in the study	
☒	☐	ChIP-seq
☒	☐	Flow cytometry
☒	☐	MRI-based neuroimaging

Antibodies

Antibodies used	β-Actin (Abcam, ab6276, 1:10000)<br>CDK2 (CST, #18048, IP=1:100, IB=1:1000)<br>Cyclin A2 (Santa Cruz, Sc-271682, IF=1:500, IB=1:100)<br>cyclin E1 (IF; Santa Cruz, sc-247, IF=1:500, IB=1:1000)

	E2F4 (CST, 40291, IP=1:1000)<br>E2F4 (Thermo Scientific, MA5 11276, IB=1:1000)<br>E2F5 (Invitrogen, PA5-85578, 1:500)<br>FOXM1 (CST, 5436, 1:500)<br>p27 (CST, 3686, 1:500)<br>RB1 (CST, 9309, 1:500)<br>RBL1 (CST, 89798, IP: 1:1000; IB: 1:500)<br>RBL2 (CST, 13610, 1:1000)<br>Vincullin (Sigma, V9131, 1:1000)<br>phospho-Histone H2A.X (gammaH2AX, CST, #9718, 1:1000)<br>phospho-RB (Ser908/811) (CST, 9308, IB=1:1000)<br>phospho-RBL2 (phospho S672) (Abcam, Ab76255, 1:1000)<br>phospho-Rb (807/811) (Alexa Fluor 647 conjugate) (Cell Signalling Technology, #8974, IF=1:2000)<br>rabbit Igg (CST, 2729, 1:2000)<br>Goat anti-rabbit-HRP conjugated secondary (CST, 7074, 1:10000)<br>Horse anti-mouse-HRP conjugated secondary (CST, 7076, 1:10000)<br>Goat anti-mouse secondary antibody, Alexa Fluor 647 (Invitrogen, A-21241, 1:1000)<br>Goat anti-rabbit secondary antibody, Alexa Fluor 647 (Invitrogen, A-21245, 1:1000)
Validation	All the antibodies used in this study are commercially available and extensively validated by the company, us, or others. Validation data is available in each of these company's website. In addition, we have confirmed the specificity of the following antibodies using siRNA-mediated knockdown and western blotting: p27 (ED Fig. 1c), FOXM1 (ED Fig. 7i), RB1 (ED Fig. 8b), p107 (RGBL1, ED Fig. 8b), p130 (RBL2, ED Fig. 8b), E2F4 (ED Fig. 8h), E2F5 (ED Fig. 8h), and cyclin A2 (ED Fig. 9c). All other antibodies were not directly validated by us but were validated by the manufacturer for the same species and application as they were used in this study.

Eukaryotic cell lines

Policy information about cell lines and Sex and Gender in Research

Cell line source(s)	MCF10A (ATCC: CRL-10317)<br>RPE-1 (ATCC: CRL-4000)<br>U2OS (ATCC: HTB-96)<br>HeLa (ATCC: CRM-CCl-2)<br>HLF (ATCC: PCS-201-013)<br>HEK293T (gift from Dr. Tobias Meyer's Laboratory at Weil Cornell Medical School, ATCC: CRL3216)<br>MCF7 (gift from Dr. Jing Huang's Laboratory at the National Cancer Institute, ATCC: HTB-22)<br>U2OS CCNA2-eYFP (gift form Dr Arne Lindqvist's Laboratory at the Karolinska institute)<br>RPE-1 CCNA2-eYFP (gift form Dr Arne Lindqvist's Laboratory at the Karolinska institute)<br>RPE-1 CCNA2dd (gift from Dr. Helfrid Hochegger's Laboratory at Sussex University)<br>MCF10A p21-/- (gift from Tobias Meyer's Laboratory at Weil Cornell Medicine)
Authentication	Cell lines purchased from ATCC were not further authenticated. MCF7 cells were authenticated by short terminal repeat (STR) analysis peformed by the Huang Lab. HEK293T, USO2 CCNA2-eYFP, RPE-1 CCNA2-eYFP, RPE-1 CCNA2dd, and MCF10A p21-/- cell lines were not authenticated.
Mycoplasma contamination	Cells used in all experiments were routinely tested for mycoplasma contimination and only mycoplasma-negative cells were used in experiments
Commonly misidentified lines<br>(See ICLAC register)	No commonly misidentified cell lines were used in the study
