# Supplementary material for: Loss of CDK4/6 activity in S/G2 phase leads to cell cycle reversal
Source: Nature. 2023 Jul 5;619(7969):363–70. doi: 10.1038/s41586-023-06274-3 (PMC10338338; doi:10.1038/s41586-023-06274-3)
Supplement: Supplementary file 3 — All raw western blots. [file 41586_2023_6274_MOESM3_ESM.pdf]

Supplementary Figure 1

Extended Data Fig. 1b

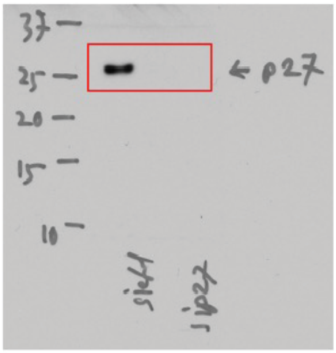

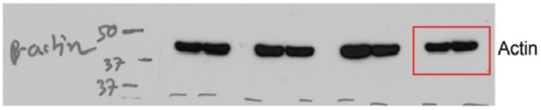

Loading control (Actin) on the same membrane

Extended Data Fig. 7b

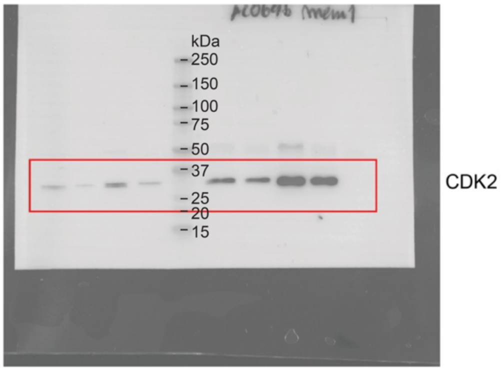

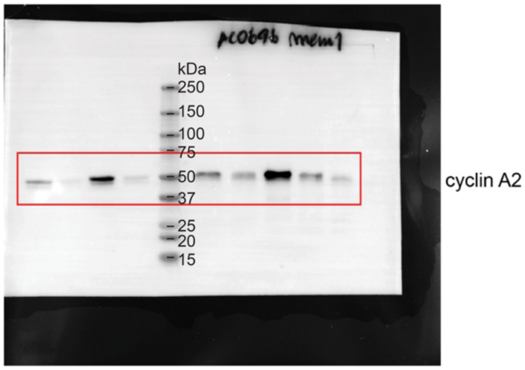

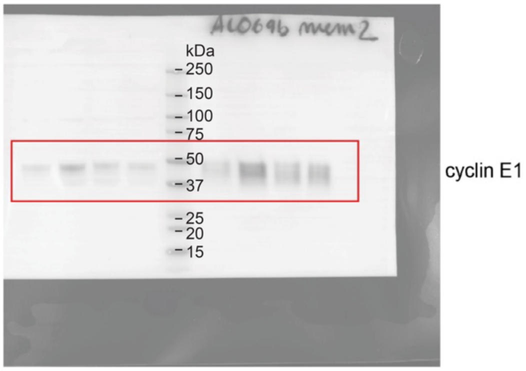

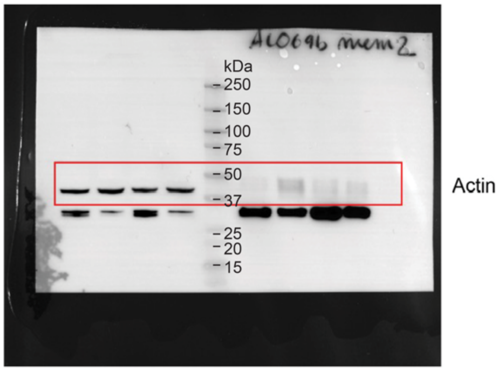

Loading control (Actin) on one of the sample membranes

Extended Data Fig. 7i

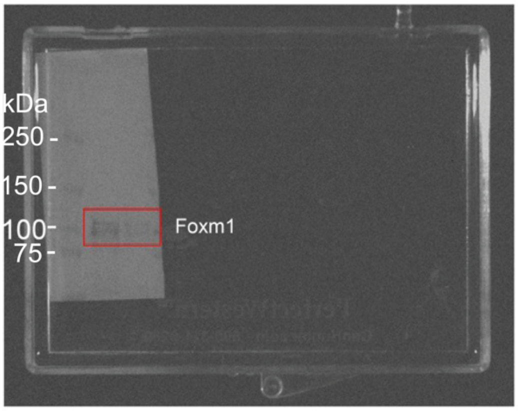

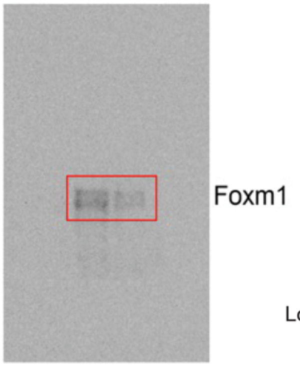

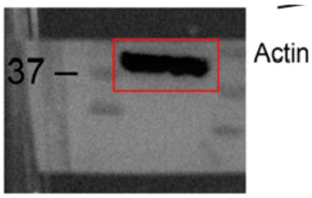

Loading control (Actin) on same membrane

Extended Data Fig. 8b

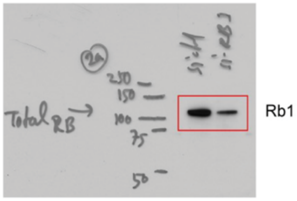

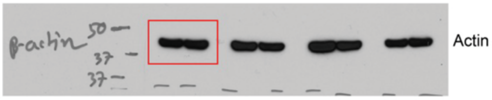

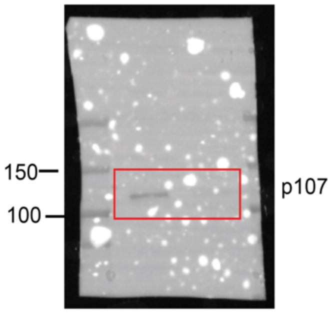

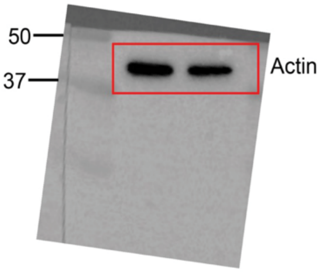

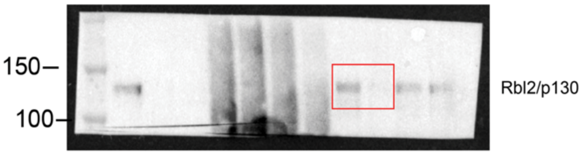

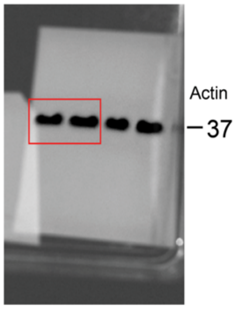

Loading control (Actin) on same membrane for each

Extended Data Fig. 8d

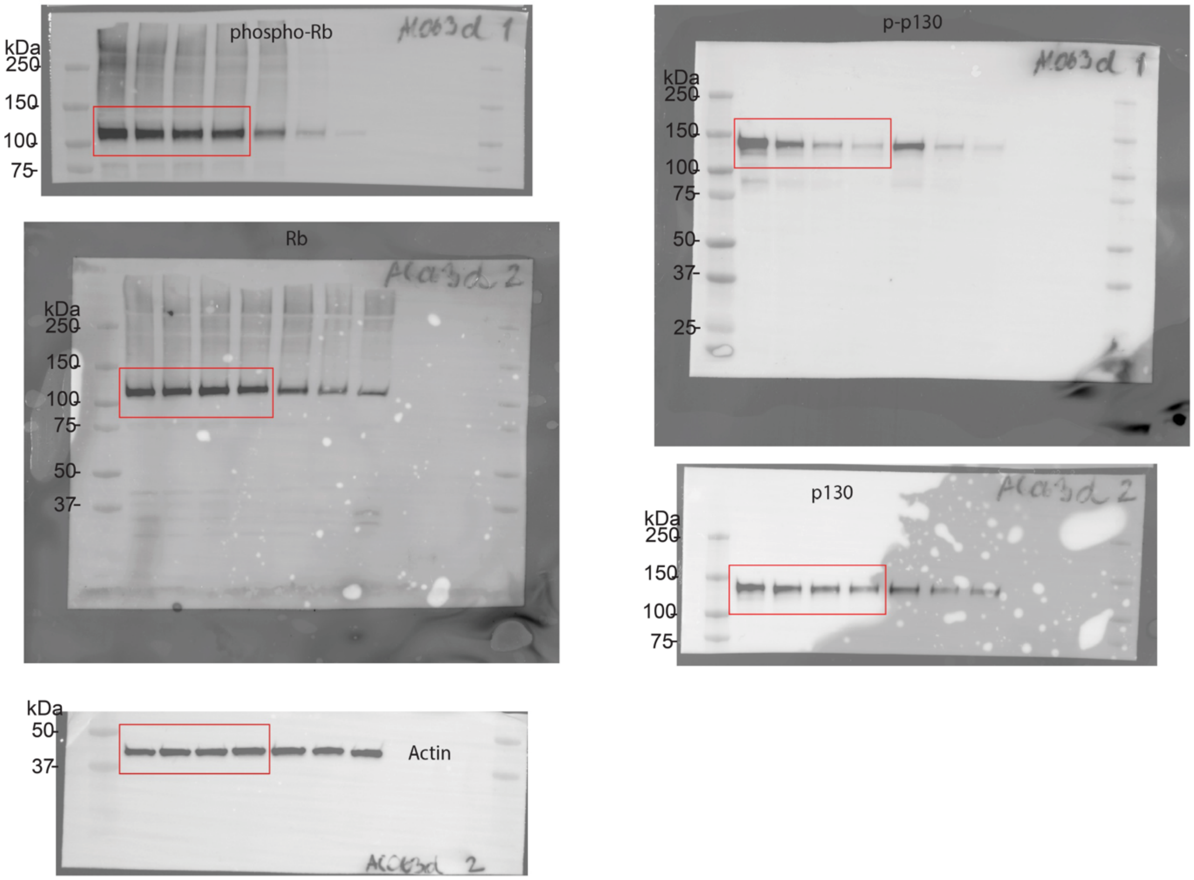

Loading control (Actin) on one of the sample membranes

Extended Data Fig. 8e

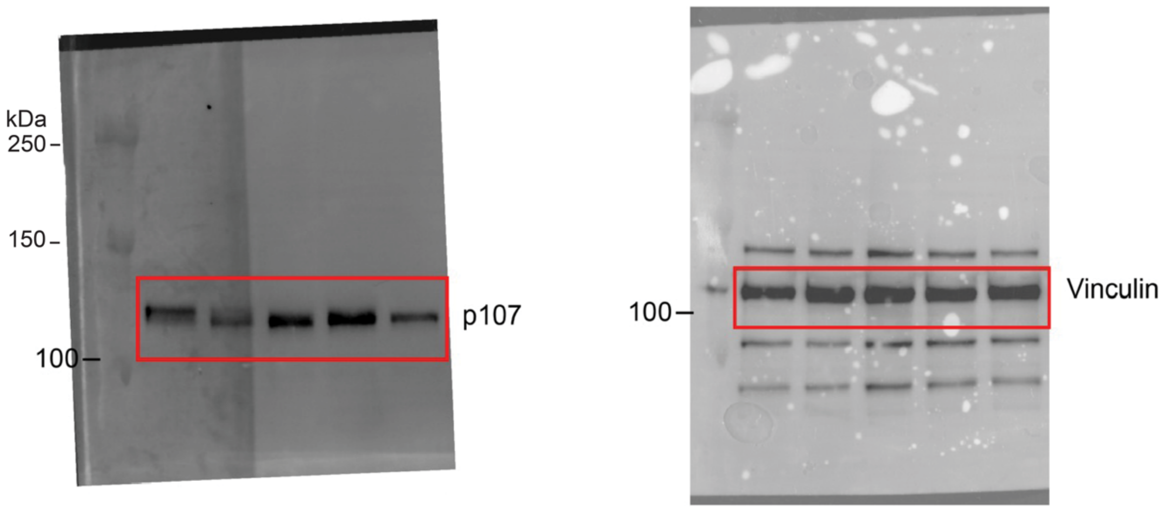

Loading control (Vinculin) on same membrane

Extended Data Fig. 8f

Membrane 1

Membrane 2

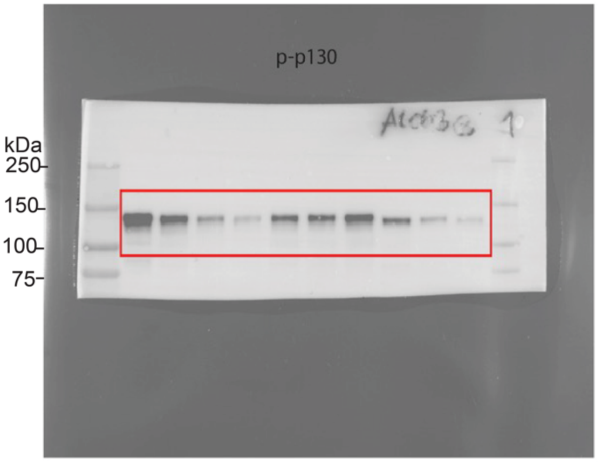

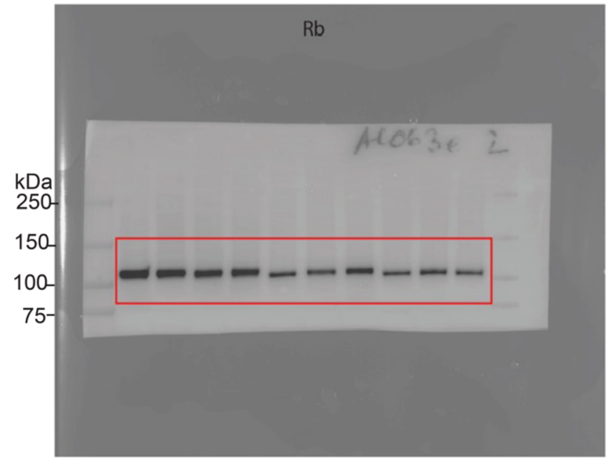

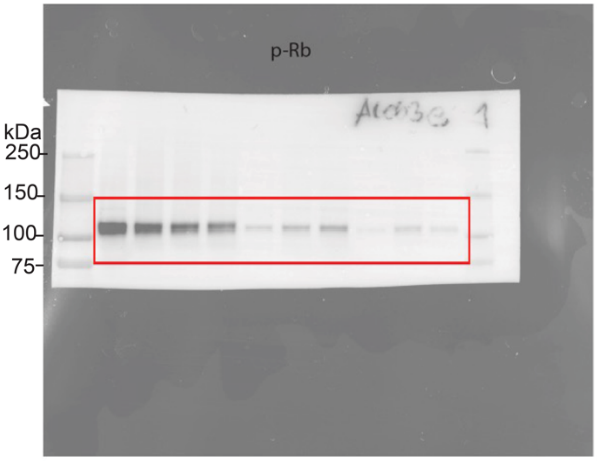

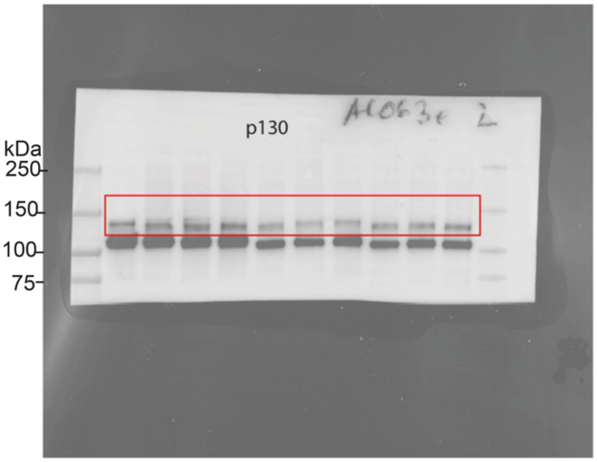

Membrane 1

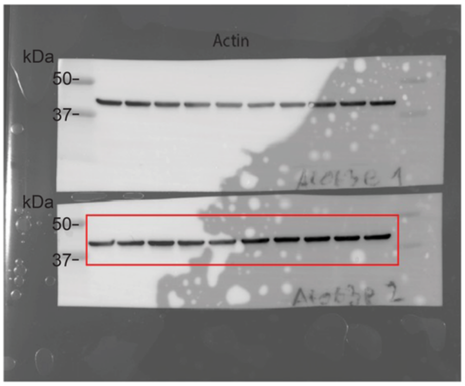

Membrane 2

Loading control (Actin) on one of the sample membranes

Extended Data Fig. 8g

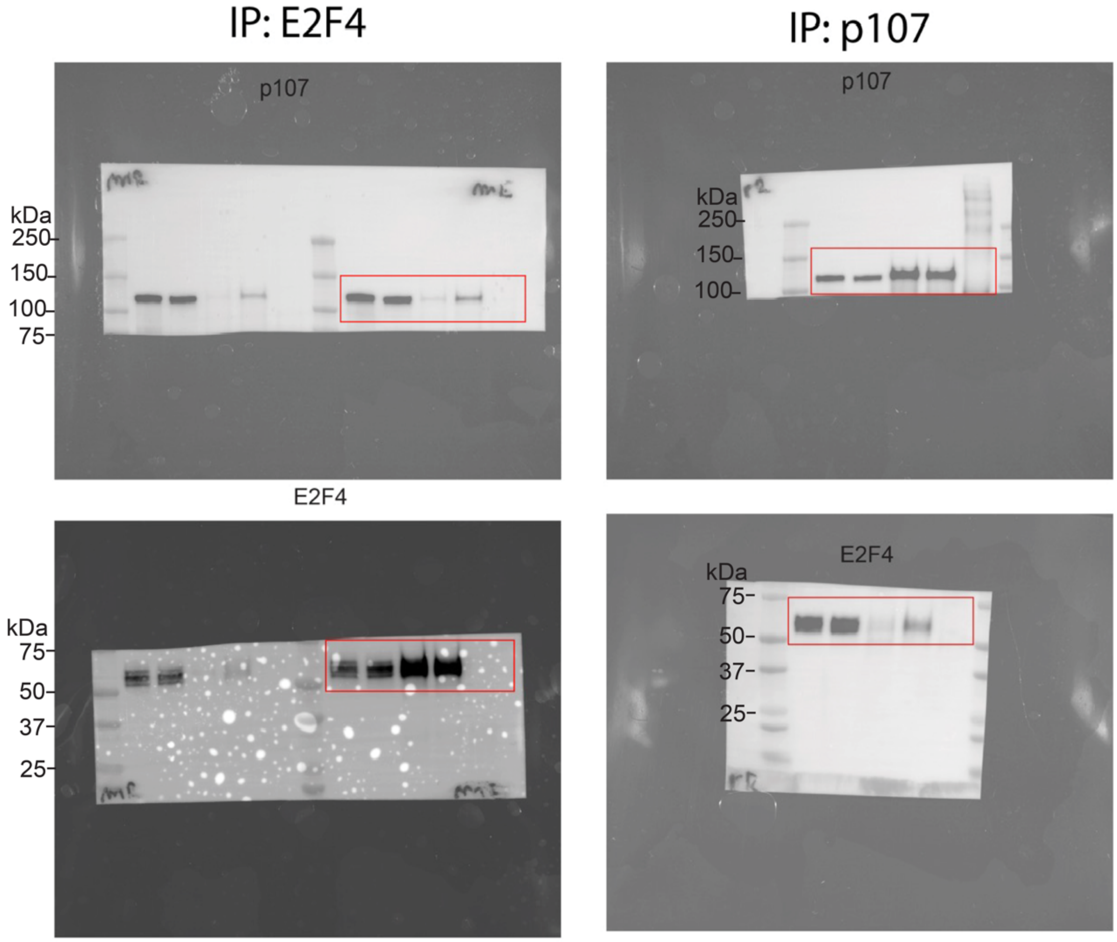

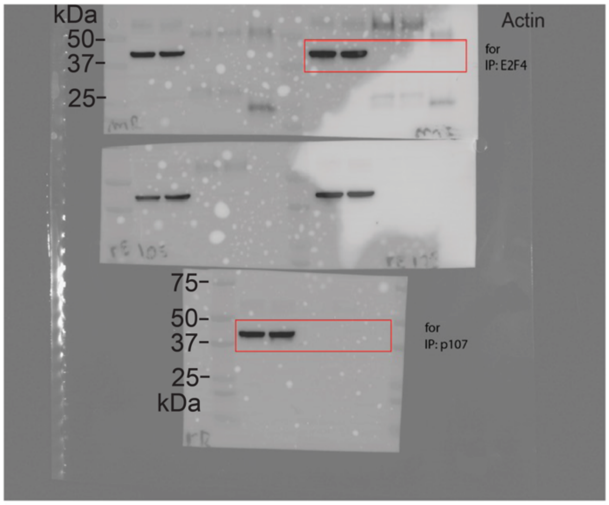

Loading control (Actin) on the same membrane

Extended Data Fig. 8h

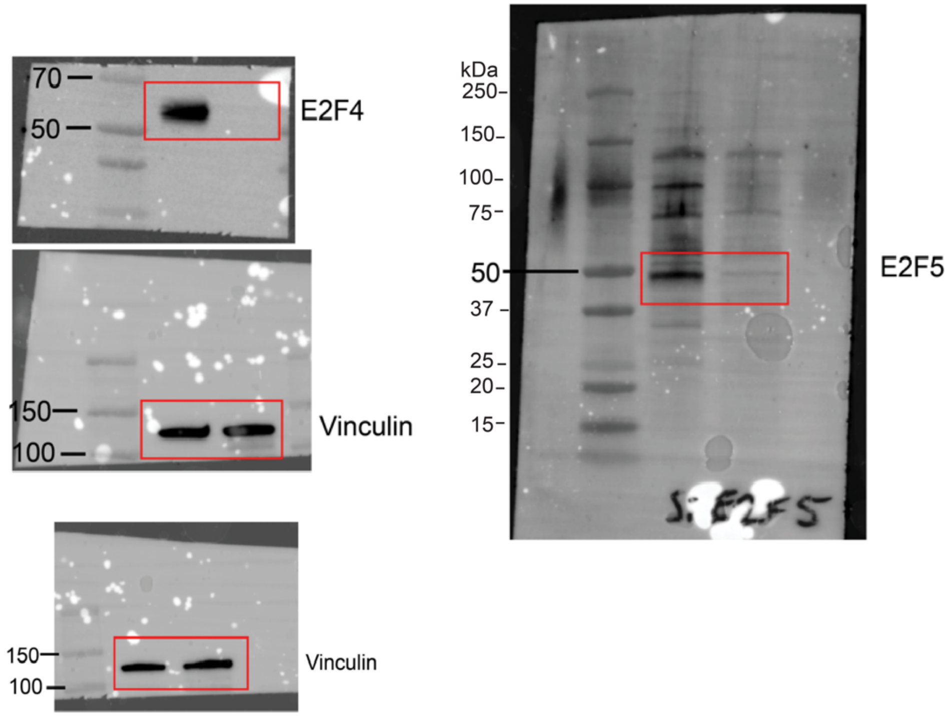

Loading control (Vinculin) on the same membrane

Extended Data Fig. 9c

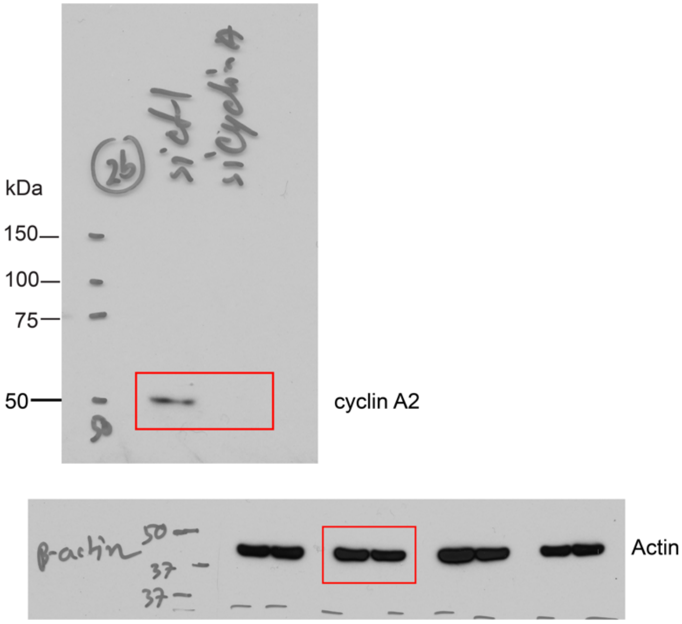

Loading control (Actin) on the same membrane
